# Associations between measurements of central blood pressure and target organ damage in high-risk patients

**DOI:** 10.1186/s40885-021-00179-x

**Published:** 2021-12-01

**Authors:** Ki-Hyun Jeon, Hack-Lyoung Kim, Woo-Hyun Lim, Jae-Bin Seo, Sang-Hyun Kim, Joo-Hee Zo, Myung-A Kim

**Affiliations:** 1 Cardiovascular center, Department of Internal Medicine, Seoul National University Bundang Hospital, Seongnam, Republic of Korea; 2 Division of Cardiology, Department of Internal Medicine, SMG-SNU Boramae Medical Center, Seoul, Republic of Korea

**Keywords:** Aortic blood pressure, Arterial pressure, Atherosclerosis, Hypertension, Pulse pressure

## Abstract

**Background:**

It is not well-known which components of central blood pressure (CBP) are more influential to target organ damage (TOD). This study aimed to determine the relationship between CBP measurements and various types of TOD in high-risk patients.

**Methods:**

A total of 148 patients who had documented atherosclerotic cardiovascular disease or its multiple risk factors were prospectively enrolled. CBP was measured by using applanation tonometry of the radial artery. The following nine TOD parameters were evaluated: left ventricular mass index, relative wall thickness, septal e′ velocity, septal E/e′, brachial-ankle pulse wave velocity, ankle-brachial index, estimated glomerular filtration rate, urine protein and obstructive coronary artery disease.

**Results:**

The mean age of the study population was 67.1 ± 9.0 years and 108 (73 %) were male. Among four CBP measurements (systolic, diastolic, mean, and pulse pressures), central pulse pressure (CPP) was associated with the largest number of TOD parameters. As CPP increased, the number of TOD increased (*P* = 0.010), but this association was not observed in other CBP measurements (*P* > 0.05 for each).

**Conclusions:**

CPP had a stronger correlation with TOD than other CBP measurements. Non-invasive CPP could be a useful indicator for predicting TOD in patients at high coronary risk.

## Background

Hypertension is one of the major risk factors for cardiovascular disease (CVD) and is a leading global burden [1, 2]. Because of ease of measurement in a practice setting, brachial blood pressure (BrBP) has been accepted as a gold standard and has been most widely used in the diagnosis, risk stratification and treatment monitoring of hypertensive patients [3, 4]. Robust evidence also suggests that baseline higher BrBP is strongly associated with future CVD outcomes [2]. Recently, the importance of central blood pressure (CBP) in the management of hypertension has been emphasized. It has been suggested that CBP is a more reliable indicator of target organ damage (TOD) and CVD risk than BrBP [5–10]. The vital organs, including the heart, brain and kidney, are more closely exposed to CBP rather than to BrBP [11–13]. Also, as blood pressure (BP) varies throughout the arterial tree, BrBP is a poor surrogate for CBP which is lower than the corresponding BrBP [14–16]. The most reliable method to measure CBP is cardiac catheterization, which can directly measure the pressure of the aortic root using a pressure-sensing catheter [17]; however, it is invasive and costly for using in routine practice. Therefore, several methods of assessing CBP using non-invasive tools have been developed, and its reliability and usefulness have been verified in several studies [15, 18].

TOD is earlier structural and functional changes in vital organs in response to long-standing high BP, such as left ventricular hypertrophy (LVH), arterial stiffening, renal impairment, and retinopathy [3]. The presence of TOD is a marker of increased CVD risk [19, 20]. Thus, evaluation of TOD is important for cardiovascular risk assessment in both subjects with and without hypertension [21]. There have been many studies on the association between CBP and TOD [11, 12, 22–26]. However, most studies focused on 1 or 2 TOD parameters, and data from systemic evaluation of the influence of CBP on various types of TOD in the same subjects have been scarce. In addition, little has been known about which measurements of CBP have a greater impact on TOD. Moreover, the role of CBP has not been well investigated in patients at high-risk. It has been suggested that TOD information is beneficial for risk stratification to determine proper treatment strategies in high-risk patients [27]. Thus, the present study was designed to investigate the relationship between CBP and TOD, using four measurements of CBP and 9 TOD parameters in patients with documented CVD or multiple risk factors.

## Methods

### Study design and population

We prospectively enrolled the study patients aged between 20 and 85 years who had documented atherosclerotic cardiovascular disease (ASCVD), including coronary artery disease and ischemic stroke, or at least two of the traditional risk factors for ASCVD (hypertension, diabetes mellitus, smoking, and obesity) from March 2017 to July 2018. In order standardize the effects of other factors influencing the CBP as much as possible and to perform a more accurate analysis, patients with following conditions were excluded: (1) recently deteriorated chest pain, dyspnea or palpitation; (2) unstable or uncontrolled BP;; (3) left ventricular ejection fraction < 50 %;; (4) valvular regurgitation or stenosis of moderate degree or greater; (5) the presence of pericardial effusion; and (6) atrial fibrillation or other uncontrolled arrhythmia. The study protocol was approved by the Institutional Review Board (IRB) of Boramae Medical Center (Seoul, Korea) (IRB No: 26-2017-55) and written informed consent was obtained from each study patient.

### Data collection

Body mass index was calculated as weight in kilograms divided by the square of height in meters (kg/m^2^). Coronary artery disease (CAD) was defined according to (1) a history of myocardial infarction or coronary revascularization, including percutaneous coronary intervention or coronary artery bypass surgery; (2) documented luminal narrowing of epicardial coronary artery more than 50 % on invasive coronary angiography or coronary computed tomographic angiography; or (3) documentation of myocardial ischemia on exercise treadmill test or single-photon emission computed tomography. Ischemic stroke was defined as a previous diagnosis: a history of focal neurological deficit that continued for > 24 h, which was confirmed by brain imaging. Hypertension was defined as previous diagnosis, the use of antihypertensive medications or a resting BP of ≥ 140/90 mmHg. Diabetes mellitus was defined as a previous diagnosis, the use of oral hypoglycemic agents or insulin or fasting blood glucose ≥ 126 mg/dL. Patients were classified as smokers if they had smoked regularly during the previous 12 months. All subjects underwent laboratory tests by sampling venous blood in the morning after overnight fasting. Blood levels of following parameters were measured by an automated enzymatic procedure: white blood cell count, hemoglobin, total cholesterol, low-density lipoprotein cholesterol and high-density lipoprotein cholesterol, triglyceride, glucose, glycated hemoglobin, creatinine, C-reactive protein, and N-terminal pro-brain natriuretic peptide (NT-proBNP). Estimated glomerular filtration rate (eGFR) was calculated using 4-component Modification of Diet in Renal Disease (MDRD) study incorporating age, race, sex, and serum creatinine level [28]. Data were also collected on current medications for CVD, including antiplatelet agents (aspirin and clopidogrel), calcium channel blockers, renin-angiotensin system blockers, beta-blockers and statins.

### Measurement of central blood pressure

Radial artery pressure waveforms and BrBP were recorded simultaneously using a fully automated device (HEM-9000AI; Omron Healthcare, Kyoto, Japan) to calculate late systolic pressure in the radial artery (SBP2) and to estimate central systolic BP (CSBP) [18]. The HEM-9000AI utilizes both oscillometer BP detection via a cuff wrap at the upper arm and tonometric measurement at the radial artery in the wrist. The device measures oscillometric signals for non-invasive BP measurement and processes the data through its computer and algorithm within the device. CBP was estimated using a regression equation with SBP2 as a major independent variable [29]. Four measurements of CBP analyzed in this study were CSBP, central diastolic BP (CDBP), central mean arterial pressure (CMAP), and central pulse pressure (CPP).

### Target organ damage parameters

The following nine parameters of TOD were assessed: (1) obstructive CAD defined as > 50 % stenosis in the major epicardial coronary arteries on computed tomographic angiography or invasive coronary angiography; (2) LVH defined as left ventricular mass index (LVMI) > 115 g/m^2^ for man and > 95 g/m^2^ for woman [30]; (3) concentric remodeling of the left ventricle defined as relative wall thickness (RWT) > 0.42 [30]; (4) diastolic dysfunction defined as septal e′ <7 cm/Sec. [31]; (5) diastolic dysfunction defined as septal E/e′ >15 [31]; (6) chronic kidney disease defined as eGFR less than 60 mL/min/1.73 m^2^; (7) proteinuria defined as albumin-creatinine ratio (ACR) > 30 mg/g in spot urine; (8) increased arterial stiffness defined as brachial-ankle pulse wave velocity (baPWV) > 1,600 cm/Secs. [32, 33]; and (9) peripheral artery disease defined as ankle-brachial index (ABI) < 0.9. The tests for TOD, including blood test, urinalysis, ABI, baPWV and echocardiography were performed at the same day of CBP measurement. Computed tomographic angiography or invasive coronary angiography for the CAD evaluation was performed within 1 week of CBP measurement.

### Statistical analysis

Continuous variables are presented as means ± standard deviation, and categorical variables are presented as number (%). Pearson correlation analysis was performed to investigate the linear association between the two parameters of CBP measurements and TOD parameters. Scatter plots were used for the demonstration of linear correlations between the two parameters. Mean values of CBP measurements between patients with or without TOD were compared using Student t-tests. Analysis of variance was used to assess the association between mean values of CBP measurements and numbers of TOD parameters. Two-sided P-values < 0.05 were considered statistically significant for all tests. All statistical analyses were performed using IMB SPSS ver. 20.0 (IBM Corp., Armonk, NY, USA).

## Results

**Fig. 1:**
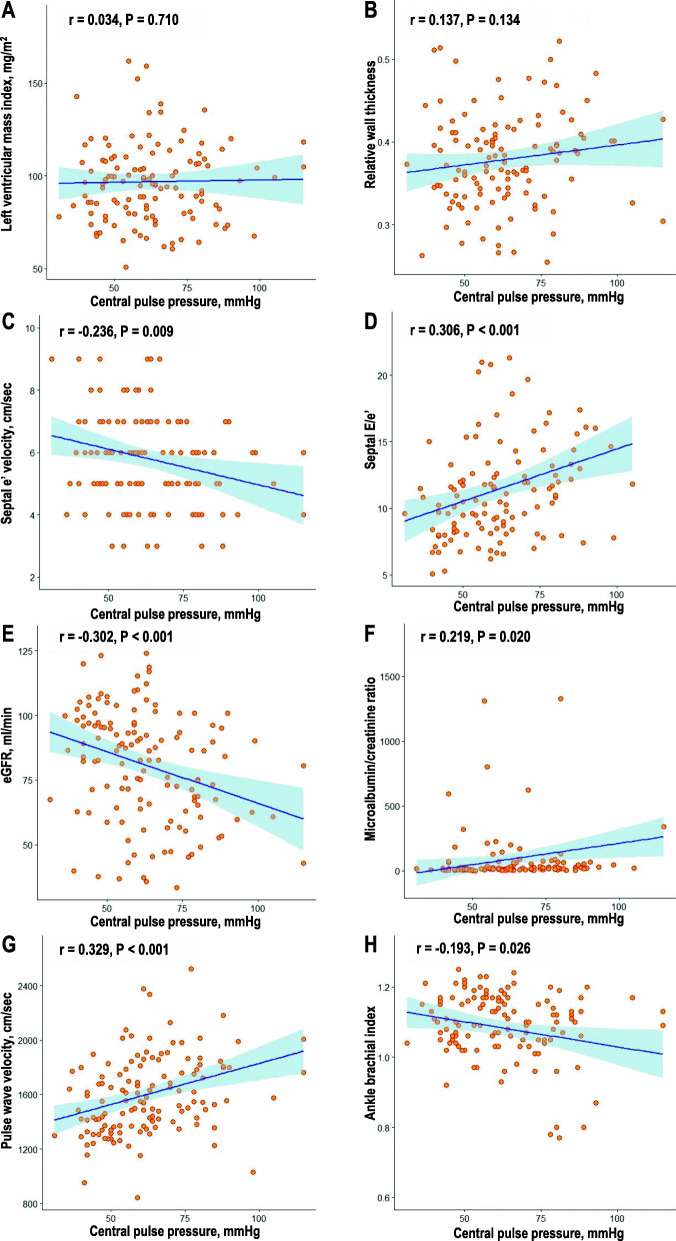
Correlations between central pulse pressure and various types of target organ damage: **A** left ventricular mass index, **B** Relative wall thickness, **C** Septal e’ velocity, **D** Septal E/e’, **E** eGFR, **F** microalbumin/creatinine ratio, **G** Pulse wave velocity, **H** Ankle brachial index. eGFR; estimated glomerular filtration rate

**Fig. 2:**
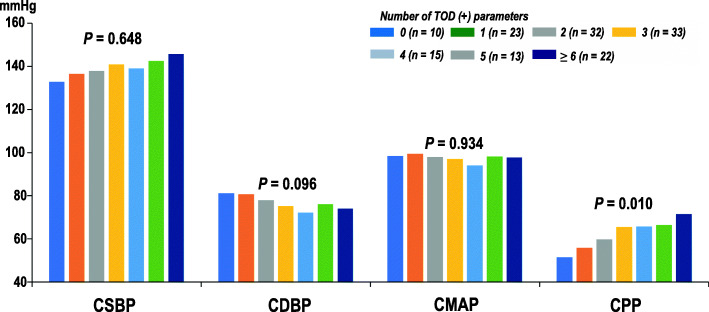
Association between central blood pressure and the number of TOD parameters. CSBP, central systolic blood pressure; CDBP, central diastolic blood pressure; CMAP, central mean arterial pressure; CPP, central pulse pressure; TOD, target organ damage

A total of 148 patients were enrolled and analyzed. The clinical characteristics of the study patients are shown in Table 1. Mean age was 67.1 ± 9.0 years, and 108 patients (73.0 %) were male. Ninety-eight patients (66.2 %) and 15 patients (10.1 %) had a history of CAD and ischemic stroke, respectively. The prevalence rates of hypertension, diabetes mellitus and current smoking in the study population were 74.3 %, 41.9 %, and 25.1 %, respectively. The results of most blood tests were within the normal range except mildly elevated blood level of NT-proBNP. Most patients were taking cardioprotective medications, such as antiplatelets (88.5 %), beta-blockers (71.3 %), renin-angiotensin system blockers (81.8 %), and statins (90.7 %). The results of BP profiles and TOD parameters are demonstrated in Table 2. Most patients had obstructive CAD (77.7 %). Table 3 shows the correlations between BP measurements and 8 TOD parameters. The presence of CAD is a binary variable and is excluded from this correlation analysis. Among eight TOD parameters, CSBP correlated with E/e′ and baPWV, CDBP with eGFR and baPWV, CMAP with baPWV, and CPP with septal e′, E/e′, eGFR, ACR, baPWV, and ABI. CPP was correlated with more TOD parameters than other CBP and BrBP measurements. LVMI and RWT were not associated with CBP measurement but associated with only brachial diastolic BP. Figure 1 shows the correlations between CPP and each value of TOD parameters. Table 4 showed the mean value of CBP measurements according to the presence of TOD. Obstructive CAD and LVH were associated with CDBP and CPP. Left ventricular (LV) diastolic dysfunction assessed by septal e′ velocity and E/e′ were associated with only CPP. LV concentric remodeling and chronic kidney disease (CKD) were not associated with either CBP measurement. Proteinuria was associated with CSBP and CPP. Increased arterial stiffness was associated with CSBP, CMAP, and CPP. Peripheral artery disease was associated with CDBP and CPP. In summary, CPP was associated with the largest number of TOD parameters. Also, CPP value increased as the number of TOD parameters increased (*P* = 0.010). Other CBP measurements did not show this association (*P* > 0.05 for each) (Fig. 2).

**Table 1:** Baseline characteristics of study population (*n* = 148)

Characteristic	Value
**Demographic finding**	
Age (yr)	67.1 ± 9.0
Male sex	108 (73.0)
Body mass index (kg/m^2^)	25.9 ± 3.5
**Comorbidity**	
Coronary artery disease	98 (66.2)
Ischemic stroke	15 (10.1)
Hypertension	110 (74.3)
Diabetes mellitus	62 (41.9)
Current smoking	38 (25.1)
**Laboratory finding**	
White blood cell count (/µL)	7,290 ± 5,240
Hemoglobin (g/dL)	14.0 ± 1.5
Cholesterol (mg/dL)	150 ± 30
Low-density lipoprotein cholesterol (mg/dL)	78.3 ± 24.1
High-density lipoprotein cholesterol (mg/dL)	47.6 ± 11.7
Triglyceride (mg/dL)	135 ± 83
Fasting blood glucose (mg/dL)	119 ± 33
Glycated hemoglobin (%)	6.35 ± 1.09
C-reactive protein (mg/dL)	0.96 ± 6.89
NT-proBNP (pg/mL)	326 ± 515
**Medication**	
Antiplatelet agent	131 (88.5)
Calcium channel blocker	73 (48.7)
Renin-angiotensin system blocker	121 (81.8)
Beta-blocker	107 (71.3)
Statin	136 (90.7)

Data are presented as mean ± standard deviation or number (%)

NT-proBNP, N-terminal pro-brain natriuretic peptide

**Table 2:** BP profiles and TOD parameters (*n* = 148)

Characteristic	Value
**BP profile**	
Right brachial systolic blood pressure (mmHg)	135 ± 16
Right brachial diastolic blood pressure (mmHg)	77 ± 10
Right brachial mean blood pressure (mmHg)	96 ± 10
Right brachial pulse pressure (mmHg)	58 ± 14
Central systolic blood pressure (mmHg)	139 ± 19
Central diastolic blood pressure (mmHg)	77 ± 10
Central mean blood pressure (mmHg)	98 ± 11
Central pulse pressure (mmHg)	69 ± 11
Central augmentation index (%)	82 ± 14
**Parameter of TOD**	
Obstructive coronary artery disease	115 (77.7)
Left ventricular mass index (g/m^2^)	97.2 ± 26.0
Relative wall thickness	0.38 ± 0.06
Septal e’ velocity (cm/sec)	5.9 ± 1.8
Septal E/e’	11.7 ± 4.4
Estimated glomerular filtration rate (mL/min/1.73 m^2^)	82.0 ± 22.1
Microalbumin creatinine ratio (mg/g)	127 ± 444
Brachial-ankle pulse wave velocity (cm/sec)	1,578 ± 314
Ankle-brachial index	1.08 ± 0.13

Data are presented as mean ± standard deviation or number (%)

BP, blood pressure; TOD, target organ damage

**Table 3:** The correlation coefficient (*r*) between BP measurements and TOD parameters

Variable	LVMI	RWT	Septal e′	E/e′	eGFR	ACR	baPWV	ABI
CSBP	0.014	0.077	–0.148	0.271^**^	–0.126	–0.041	0.416^**^	–0.142
CDBP	–0.125	–0.011	–0.011	–0.011	0.192^*^	–0.096	0.247^**^	0.149
CMAP	–0.067	0.038	–0.007	0.149	0.045	–0.091	0.379^**^	0.007
CPP	0.034	0.137	–0.236^**^	0.306^**^	–0.302^**^	–0.219^*^	0.329^**^	–0.193^*^
BrSBP	–0.011	0.065	–0.096	0.267^**^	–0.090	0.032	0.398^**^	–0.151
BrDBP	–0.233^**^	–0.015^*^	0.131	–0.015	0.139	–0.096	0.218^*^	0.047
BrMAP	–0.155	0.022	0.032	0.128	0.043	–0.048	0.350^**^	–0.043
BrPP	0.148	0.083	–0.197^*^	0.308^**^	–0.196^*^	0.099	0.291^**^	–0.203^*^

BP, blood pressure; TOD, target organ damage; LVMI, left ventricular mass index; RWT, relative wall thickness; eGFR, estimated glomerular filtration rate; ACR, albumin-creatinine ratio; baPWV, brachial-ankle pulse wave velocity; ABI, ankle-brachial index; CSBP, central systolic blood pressure; CDBP, central diastolic blood pressure; CMAP, central mean arterial pressure; CPP, central pulse pressure; BrSBP, brachial systolic blood pressure; BrDBP, brachial diastolic blood pressure; BrMAP, brachial mean arterial pressure; BrPP, brachial pulse pressure

^*^P < 0.05, ^**^P < 0.001

**Table 4:** Mean values of central blood pressure measurements according to the presence of TOD

Variable	TOD (+)	TOD (–)	P-value
**Obstructive CAD**	(+) (*n* = 104)	(–) (*n* = 30)	
CSBP, mmHg	139 ± 19	139 ± 18	0.881
CDBP, mmHg	75.4 ± 9.7	81.1 ± 9.7	0.007
CMAP, mmHg	96.8 ± 11.3	100.5 ± 11.3	0.115
CPP, mmHg	64.1 ± 17.1	56.0 ± 14.3	0.036
**LVMI**	> 115 g/m^2^ for male and > 95 g/m^2^ for female (*n* = 91)	≤ 115 g/m^2^ for male and ≤ 95 g/m^2^ for female (*n* = 31)	
CSBP, mmHg	140 ± 20	138 ± 19	0.770
CDBP, mmHg	72.8 ± 9.9	77.2 ± 9.8	0.031
CMAP, mmHg	95.1 ± 10.7	97.7 ± 11.7	0.294
CPP, mmHg	68.1 ± 20.0	61.3 ± 15.4	0.041
**RWT**	> 0.42 (*n* = 24)	≤ 0.42 (*n* = 98)	
CSBP, mmHg	142 ± 23	138 ± 18	0.311
CDBP, mmHg	76.0 ± 10.8	76.1 ± 9.8	0.976
CMAP, mmHg	98.2 ± 12.9	96.7 ± 11.1	0.565
CPP, mmHg	66.7 ± 21.0	61.9 ± 15.6	0.307
**e′ velocity**	< 7 cm/sec (*n* = 105)	≥ 7 cm/sec (*n* = 17)	
CSBP, mmHg	140 ± 20	132 ± 14	0.113
CDBP, mmHg	75.6 ± 10.1	78.9 ± 8.6	0.216
CMAP, mmHg	97.1 ± 11.8	96.7 ± 9.7	0.888
CPP, mmHg	64.4 ± 17.1	53.1 ± 10.6	0.010
**E/e′**	> 15 (*n* = 25)	≤ 15 (*n* = 97)	
CSBP, mmHg	143 ± 23	137 ± 18	0.173
CDBP, mmHg	73.8 ± 11.9	76.7 ± 9.4	0.192
CMAP, mmHg	97.1 ± 14.5	97.0 ± 10.6	0.967
CPP, mmHg	70.1 ± 16.5	60.0 ± 16.5	0.015
**eGFR**	< 60 mL/min/1.73 m^2^ (*n* = 27)	≥ 60 mL/min/1.73 m^2^ (*n* = 107)	
CSBP, mmHg	143 ± 19	138 ± 19	0.330
CDBP, mmHg	74.5 ± 8.6	77.3 ± 10.2	0.188
CMAP, mmHg	97.1 ± 10.9	97.8 ± 11.5	0.791
CPP, mmHg	67.8 ± 16.7	61.4 ± 16.5	0.085
**ACR**	> 30 mg/g (*n* = 43)	≤ 30 mg/g (*n* = 78)	
CSBP, mmHg	144 ± 19	136 ± 18	0.034
CDBP, mmHg	76.2 ± 10.0	76.5 ± 9.7	0.877
CMAP, mmHg	98.7 ± 11.2	96.6 ± 11.0	0.303
CPP, mmHg	67.8 ± 18.5	60.1 ± 14.8	0.015
**baPWV**	> 1,600 cm/sec (*n* = 57)	≤ 1,600 cm/sec (*n* = 77)	
CSBP, mmHg	145 ± 19	135 ± 18	0.003
CDBP, mmHg	77.3 ± 10.5	76.3 ± 9.6	0.578
CMAP, mmHg	99.9 ± 11.9	95.9 ± 10.7	0.043
CPP, mmHg	68.0 ± 16.6	58.8 ± 15.7	0.001
**ABI**	< 0.9 (*n* = 9)	≥ 0.9 (*n* = 125)	
CSBP, mmHg	145 ± 24	139 ± 19	0.353
CDBP, mmHg	67.6 ± 11.4	77.4 ± 9.6	0.004
CMAP, mmHg	93.6 ± 14.6	97.9 ± 11.1	0.277
CPP, mmHg	77.8 ± 17.3	61.7 ± 16.2	0.005

TOD, target organ damage; CAD, coronary artery disease; CSBP, central systolic blood pressure; CDBP, central diastolic blood pressure; CMAP, central mean arterial pressure; CPP, central pulse pressure; LVMI, left ventricular mass index; RWT, relative wall thickness; eGFR, estimated glomerular filtration rate; ACR, albumin-creatinine ratio; baPWV, brachial-ankle pulse wave velocity; ABI, ankle-brachial index

## Discussion

Our result, using four CBP measurements and nine TOD parameters, showed that each CBP measurement were related to different TOD parameters in patients with ASCVD or its multiple risk factors. Among four CBP measurements (CSBP, CDBP, CMAP, and CPP), CPP was the best correlate with TOD. It also revealed that the higher CPP was, the more target organs were damaged.

Vital organs, such as the heart, kidneys, and brain, are more directly exposed to CBP rather than to BrBP. In this study, CPP was correlated with the most indices of TOD (6/8), although both CBP and BrBP parameters could predict TOD well. There is a theoretical background that central aorta is closer to major organs, so CBP’s influence is greater than BrBP [34, 35]. CBP represents the true pressure load imposed on the heart, kidney and brain, and flow derived by CBP influences the local flow into these vital organs [13]. Therefore, CBP could be better correlated with cardiovascular prognosis and TOD than BrBP [9]. In the Strong Heart Study, CBP was more strongly associated with future cardiovascular events than BrBP in disease-free individuals [5]. Other studies have shown that CBP lowering may be responsible for LVH regression and the improvement in cardiovascular prognosis beyond BrBP lowering [8, 36]. These findings support the hypothesis that CBP-lowering drugs may be more effective than BrBP-targeting ones [8].

It is not well-known which CBP measurements are clinically relevant. A few studies have emphasized the importance of CPP, as it was associated with the risk of TOD and ASCVD. Jankowski et al. [37] showed that CPP and pulsatility of the central aorta were the most important factors related to CAD rather than BrBP, and Central Aortic Pressure and Clinical Outcomes (CAFÉ) study also revealed that CPP may be a main determinant of clinical outcomes [8]. Madhavan et al. [38] demonstrated that the wider pulse pressure (PP) was identified as a predictor of myocardial infarction. In line with these studies, our findings also showed that CPP was most important factor predicting TOD among CBP measurements.

Possible mechanisms explaining the association between CPP and TOD can be suggested. Elevation of PP is both a cause and a result of arterial damage and atherosclerosis. Degenerative changes in the aortic wall and arterial tree by aging increase stiffness of the aorta, and lead to an increase in PP [39]. With repeated exposure to increased CPP, the arteries are directly damaged and arteriosclerosis progresses [21]. Progressive aortic stiffness increases systolic BP and decreases diastolic BP, which makes CPP wider [40]. Therefore, CPP is an indicator of aortic stiffness. Patients with increased aortic stiffness share common cardiovascular risk factors of TOD, such as aging, high BP, hyperglycemia, and dyslipidemia [41]. In addition, increased systolic BP cause LVH, and low diastolic BP is associated with decreased coronary blood flow [42].

Risk stratification and early aggressive management for high-risk patients is important for preventing future cardiovascular events and for reducing morbidity and mortality. The present study revealed that CBP, especially CPP, is valuable in the prediction of TOD. Measurement of CBP using radial artery tonometry, which is non-invasive and well-validated, could be useful for the risk stratification of high-risk patients. This study could also be a hypothesis-generating trial for further investigations into the development of novel medications lowering CPP or using CPP as a monitoring tool.

Some limitations are present in this study. First, because cardiovascular outcomes, such as myocardial infarction or mortality, were not investigated, we could not conclude whether CPP was related to long-term prognoses of patients. However, as TOD is a well-known factor closely related to worse cardiovascular outcomes [19, 20], CPP might be a prognostic factor of future cardiovascular events. Longitudinal studies with a larger sample size are warranted. Secondly, due to the relatively small number of study patients, we were able to show only weak linear correlations between CBP and the parameters of TOD. There was also a possibility that associations between some other CBP measurements and TOD did not reach statistical significance. For similar reason, the superiority of CBP over BrBP could not be proved in this study, however, the purpose of our study is not to compare CBP and BrBP. Thirdly, the duration or risk factors of ASCVD could have had an effect on CBP measurements and TOD; however, our data did not provide information on this. Finally, as our data were collected from Korean patients at high coronary risk, it should be cautious in applying them to other ethnic groups of patients.

## Conclusions

CPP had a stronger correlation with TOD than other CBP measurements in patients with ASCVD or multiple risk factors. Non-invasive measurement of CPP may be a useful tool for risk stratification of high-risk patients.

## Data Availability

The datasets used and/or analyzed during the current study are available from the corresponding author on reasonable request.

## Abbreviations

ABI: Ankle-brachial index
ACR: Albumin-creatinine ratio
ASCVD: Atherosclerotic cardiovascular disease
baPWV: Brachial-ankle pulse wave velocity
BP: Blood pressure
BrBP: Brachial blood pressure
BrDBP: Brachial diastolic blood pressure
BrMAP: Brachial mean arterial pressure
BrPP: Brachial pulse pressure
BrSBP: Brachial systolic blood pressure
CAD: Coronary artery disease
CBP: Central blood pressure
CDBP: Central diastolic blood pressure
CKD: Chronic kidney disease
CMAP: Central mean arterial pressure
CPP: Central pulse pressure
CSBP: Central systolic blood pressure
CVD: Cardiovascular disease
eGFR: Estimated glomerular filtration rate
LV: Left ventricular
LVH: Left ventricular hypertrophy
LVMI: Left ventricular mass index
MDRD: Modification of diet in renal disease
NT-proBNP: N-terminal pro-brain natriuretic peptide
PP: Pulse pressure
RWT: Relative wall thickness
TOD: Target organ damage
